# A qualitative study to explore the perception and behavior of patients towards diabetes management with physical disability

**DOI:** 10.1186/s13098-017-0257-6

**Published:** 2017-07-24

**Authors:** Syed Wasif Gillani, Syed Azhar Syed Sulaiman, Mohi Iqbal Mohammad Abdul, Sherif Y. Saad

**Affiliations:** 1 Clinical and Hospital Pharmacy Department, College of Pharmacy, Taibah University, Medina, Kingdom of Saudi Arabia; 2 Pharmacotherapy Research Group, Puncak Alam, Malaysia; 3 School of Pharmaceutical Sciences, Universiti Sains Malaysia (USM), Pinang, Malaysia; 4 College of Pharmacy, University of Philippines, Quezon City, Philippines; 5 Cancer Biology Department, National Cancer Institute, Cairo University, Kasr Al-Aini Street, Cairo, Egypt

**Keywords:** Patient education, Counseling, Disease understanding, Diabetes mellitus, Qualitative study

## Abstract

**Background:**

This study aimed to determine self-monitoring practices, awareness to dietary modifications and barriers to medication adherence among physically disabled type 2 diabetes mellitus patients.

**Methods:**

Interview sessions were conducted at diabetes clinic—Penang general hospital. The invited participants represented three major ethnic groups of Malaysia (Malay, Chinese and Indians). An open-ended approach was used to elicit answers from participants. Interview questions were related to participant’s perception towards self-monitoring blood glucose practices, Awareness towards diet management, behaviour to diabetes medication and cues of action.

**Results:**

A total of twenty-one diabetes patients between the ages 35–67 years with physical disability (P1–P21) were interviewed. The cohort of participants was dominated by males (n = 12) and also distribution pattern showed majority of participants were Malay (n = 10), followed by Chinese (n = 7) and rest Indians (n = 4). When the participants were asked in their opinion what was the preferred method of recording blood glucose tests, several participants from low socioeconomic status and either divorced or widowed denied to adapt telemonitoring instead preferred to record manually. There were mixed responses about the barriers to control diet/calories. Even patients with high economic status, middle age 35–50 and diabetes history of 5–10 years were influenced towards alternative treatments.

**Conclusions:**

Study concluded that patients with physical disability required extensive care and effective strategies to control glucose metabolism.

**Practice implication:**

This study explores the patients’ perspectives regarding treatment management with physical disability.

## Background

The most recent report by International Diabetes Federation *Diabetes Atlas* estimates that there are currently 387 million people living with diabetes globally in 2014, a 105% increase from its last report in 2011 with most people living in the western pacific [1]. Recent systematic analysis study on global burden disease analysed data from health examination surveys and epidemiological studies included data from 2.7 million participants and 370 country-years reports that a total of 347 million adults are living with diabetes worldwide [2]. It is estimated that by 2030 a total of 439 million people will suffer from diabetes mellitus, which represents approximately 7.7% of the global adult population aged 20–79 years [3].

Patients with medication non adherence may failed to achieve optimal therapeutic outcomes [4–6]. Physiologically hemoglobin A1c inversely related to diabetes medication adherence [6]. Several studies have determined the link between medication non adherence with higher diabetes related complications, inpatient and emergency department utilizations [3, 7]. There are several factors effecting the glycemic control and patient adherence to the treatment plan [8, 9]. To achieve target glycemic control, patients needed to follow multiple care models including self-monitoring blood glucose (SMBG), Dietary modifications, exercise, improve diabetes medication knowledge and medication adherence [5, 7].

Disability is a key indicator implicating both overall morbidity and success of public health efforts to compress the period of morbidity among geriatrics for the overall population. Disabilities are more prevalent among diabetics than among those without diabetes. Physical inactivity, obesity, peripheral arterial disease, neuropathy, coronary heart disease and depression contribute strongly to higher disability risk among diabetic persons. Better management of glycaemia and reduction of risk factors for cardiovascular disease provide long-term prevention of disability. Preventing disability will likely depend on a combination of secondary and tertiary prevention along with diabetes prevention [8]. Common disabling conditions among people with diabetes in the United States include arthritis that limits physical activity, depression, hearing loss, peripheral neuropathy and visual impairment that limits ability to read regular print [9]. Improving behaviors of patient and clinician regarding close monitoring of disease control parameters and timely treatment adjustments might improve quality of life among patients with multiple comorbidities and complex health care needs [10]. Diabetes-induced disability rate is increasing due to the fact that the vast majority of diabetics are living longer. Due to poor medication adherence among diabetic subjects contribute to exaggerated health cost. Diabetes associated disabilities contribute to great extend poor adherence to prescribed medications, since a huge number among diabetics at the time of diagnosis, have experienced disabilities [11]. Mortality among diabetics has now been postponed to older age in most cases; however disability and health loss due to diabetes is increasing, particularly in the older population [12]. The complexity of self-care often increases as diabetic subject is growing older. Since eyesight, hearing, fine motor skills and memory processes are altering with time resulting in a great impact on the individual’s ability to comply with self-care practices [13].

Physical disability and cognitive impairments are the major barriers to achieve optimal glycemic control and medication adherence. Somehow the research community ignored to explore the patients behavior to self-care practices and medication adherence with physical disability. Thus this study aimed to determine self-monitoring practices, awareness to dietary modifications and barriers to medication adherence among physically disabled type 2 diabetes mellitus patients.

## Methods

### Research design

Qualitative method explores the understanding of participants’ behavior “how and why people respond to disease management practices”. In addition, such methods also provide comprehensive answers to diverse questions from patient oriented barriers to drug related problems. The qualitative interview has the flexible nature of exploration that is advantageous to the researcher investigating knowledge, perception and barriers to respond.

### Setting and participants

Interview sessions were conducted at diabetes clinic—Penang general hospital (2016–2017). The invited participants represented three major ethnic groups of Malaysia (Malay, Chinese and Indians).

Eligibility criteria: patient with physical disability (amputee arm and/or leg), diabetes type II mellitus and aged 18 years or above. Recruitment was performed in suggestion with physicians attending patients at diabetic clinic (6-months, systemic random sampling). Patients with cancer, pregnancy, inflammatory disorder or cognitive impairment (dementia etc.) were excluded.

Participants did not face any challenges when answering interview questions during the interview session as the questions used were simple and straightforward without the use of medical jargons.

### Assessment tool

A semi-structured interview guide was used to conduct the study (Table 1). An open-ended approach was used to elicit answers from participants. Interview questions were related to participant’s perception towards self-monitoring blood glucose practices, Awareness towards diet management, behaviour to diabetes medication and cues of action. General probing was used during the interview sessions to facilitate questions (Can you explain further? What about your opinion on this? Can you further clarify etc.).

**Table 1:** Interview guide

Discussion topic	Examples of specific probes
Perception towards self-monitoring blood glucose practices	In your opinion what is the preferred method of recording blood glucose reading?
	Do you think self-monitoring of blood glucose useful for diabetes management?
	What stops people for self-care practices?
Awareness towards diet management	In your opinion what are the strategies to control diet?
	Before you diagnosed (diabetes), have you heard of calorie counting?
Behavior to diabetes medication	What type of experiences with diabetes medication usually reduces the people adherence?
	Do you aware of other beliefs (lay beliefs) in people that influence the diabetes management?
	Have you heard of alternative medicines for diabetes?
Cues to action	What would you like to suggest improving diabetes management behavior among other diabetes patients?

### Tool development and validation

The interview probe guide was first developed after extensive literature search [10–13] and then discussed with the experts from both academic and practice oriented personnel. The purpose to conduct this process was to merge healthcare providers’ prospective coherently with interview specific probes. This will interest public health experts and endocrinologist to follow-up with research findings and improve future practices. A pilot study was conducted to pre-test the interview guide but the data is neither presented in this manuscript nor added to final analysis (sample size of pilot study—n = 8).

### Interview process

Due to the large amount of participants who are from the Malay ethnic group interviews were conducted in local Malaysian language (n = 18). Interviews were conducted in English where language barrier was not a concern (n = 3). The back translate method is used to report the quotes of the local Malaysian language interviews to make sure the concepts translated properly. Three research assistants, one from each ethnic (Malay, Chinese, Indian) were trained to conduct the interviews. On average interview sessions were approximately forty minutes in length (30–60 min). The principle investigator facilitated all the interview sessions with research assistants and also documented field notes. Prior to interview patients’ demographic and disease data was collected by a structured questionnaire attached with patient information sheet and consent form.

### Ethical considerations

Research ethics approval was acquired prior to the commencement of the study, from Clinical Research Committee (CRC), Ministry of Health Malaysia (NMRR-10-776-6941). Informed consent was obtained from all the participants in either English or Malay languages. Verbal consent was considered from those unable to read or write.

### Data analysis/evaluation

All the interviews were audiotaped for verbatim transcriptions. All the interviews were transcribed by principle investigator to avoid bias. The transcripts were then verified for accuracy by relevant participants and proceed for analysis after approval. The principle investigator recorded the raw data thematically and then the themes were discussed with other expert independent researchers to ensure the reliability and trustworthiness [14]. Each transcript was repeatedly read by three independent experts to identify the common theme. Emergent theme was then discussed among all the authors to refine the analysis. The investigators continued (and not concluded) interviews until theoretical saturation was achieved, when subsequent interview not produce any new information (saturation + 3 formula applied) [15].

## Results and findings

A total of twenty-one diabetes patients between the ages 35–67 years with physical disability (P1–P21) were interviewed. The cohort of participants was dominated by males (n = 12) and also distribution pattern showed majority of participants were Malay (n = 10), followed by Chinese (n = 7) and rest Indians (n = 4). Majority of them were married (n = 9) and also moderate socioeconomic status (n = 10). A total of eight participants had diabetes history of 11–15 years and about half of the participants (n = 10) reported oral treatment for diabetes. The demographic and clinical characteristics of participants are summarized in Table 2. All the patients were asked about regular monitoring/follow-up to physician before the interview and majority of the participants (n = 18) reported either missed appointments or forget follow-up monitoring.

**Table 2:** Demographic and clinical characteristics of participants (n = 21)

Characteristics	N	%
Age (mean ± SD) = 45.89 ± 7.51 years		
Range		
18–30	2	9.5
31–40	5	23.8
41–50	7	33.4
51–60	4	19.0
≥61	3	14.3
Gender		
Male	12	57.1
Female	9	42.9
Ethnicity		
Malay	10	47.6
Chinese	7	33.4
Indians	4	19.0
Educational status		
Primary	7	33.4
Secondary	6	28.5
College	5	23.8
Tertiary	3	14.3
Socioeconomic status		
Low (<RM^a^ 1000/month)	4	19.0
Moderate (RM 1000–3000/month)	10	47.6
High (RM 3100>/month)	7	33.4
Duration of diabetes (years)		
Less than 5	3	14.3
5–10	7	33.4
11–15	8	38.1
16–20	2	9.5
More than 20	1	4.7
Marital status		
Single	2	9.5
Married	9	42.9
Divorced	6	28.5
Widowed	4	19.1
Treatment mode		
Oral anti-hyperglycemic drugs	10	47.6
Insulin	6	28.5
Oral and insulin combination	5	23.9
Physical disability		
Amputate arm/hand	12	57.1
Amputate leg/foot	9	42.9

^a^Ringgit Malaysia

### Perception towards self-monitoring blood glucose practices

When the participants were asked in their opinion what was the preferred method of recording blood glucose tests, several participants from low socioeconomic status and either divorced or widowed denied to adapt telemonitoring instead preferred to record manually.

*“I (prefer to) manually record. I do not understand how to use a telephone especially opening (applications and other function on the telephone). To me manual (recording) is easier”…*… (P10)

However, participants from moderate or high economic status and either single or married showed positive perception/willingness to adapt technology based monitoring.

*“I am an old person I like it to be (hand) written. Anyway as long as someone shows me how to do it I can do it (electronic monitoring). Of course it’s easier because you bring your hand phone everywhere you go”….* (P18)

At the same time, participants also claimed that use of technology would be portable to carry along and helped them to record easily, also provide detailed log of all the tests to attending physicians and reduces dependency to others.

*“(I prefer the) digitals way (telemedicine). Everyday you can see it in your digital way in the software (digital diary) so (there is) no need to record like manually. Sometime(s) even (if) you record manually the paper (is placed) wherever (and will go) missing. (With telemedicine) you have a backup. Due to (limited mobility) I am dependent on family members for (regular check*-*up), so this electronic log (will help my physician) to track down my performance”…..* (P21)

*“I think, It’s useful to me as an indication (of my sugar control). I prefer that I can use it to check my blood sugar (levels and so I can study how this medication effect(s) my glucose (levels). Also this (reduces my dependency) to family members”*… (P6)

Barrier to self-care practices; majority of participants with age >40 years and diabetes history >11 years showed concern about financial conflicts, however patients age >60 years either dependent to other caregiver for blood glucose monitoring or usually reluctant to self-monitoring and limited with the experience of diabetes related symptoms.

*“Self*-*monitoring is okay but sometimes*-*financial conflict (unable to buy sticks for glucometer) let me forget about checking my sugar for months… then suddenly I few symptoms (hyperglycaemic or hypoglycaemic) pops*-*up and I remember to continue my sugar monitoring”…* (P1)

*“Well what (I can say), I am (afraid) of blood, so I cant monitor (my self) sugar… sometimes my son (when free) check the sugar… Usually (twice or three) times per month.. but sometimes I feel (dizzy) so I asked him to check (blood sugar)”*… (P7)

### Awareness towards diet management

When the participants were asked before you diagnosed (diabetes), have you heard of calorie counting, majority of the participants regardless of age, marital status and years of diabetes history were denied.

*“We do not know (about calories) we just eat whatever we fancy regardless how how much calorie is in the food”….* (P15)

There were mixed responses about the barriers to control diet/calories.

*“It is not hard to control (our diet but) sometimes we (do not want to) waste (food) so we will finish (up any left overs). Sometimes your wife might be stressed at work and (when you) come back and say ‘What is this (kind of food)!” then it will become a big issue. (Do you) understand?”…* (P8)

*“If we cook separately) it can affect our relationship (with or families). When I do it like that (insisting on eating healthy food) your (there will be) a rift in your family(ies) relationship so sometimes we do not follow (our diet) that strictly because dinner time is the only time (for a) family gathering so sometimes we will eat out”*… (P3)

Participants have mentioned several strategies to control diet but it seems ineffective. Reduction in food intake especially carbohydrates as well as reducing food intake was reported. Even so, some participants remain hesitant to completely changing their diets in order to maintain a healthy relationship among their family members. Hence compromises are made. Eventually participant’s diets are not controlled.

*“I have my wife (who does the cooking). I’m living in a standard family (of) more than six adult people and more than three children (we) have to cook a lot and then I will have to cook separately”….* (P2)

*“I change everything (diet) because rice is very bad. (I will eat) rice maybe two (to) three time(s) a day (week) only so (instead) I (will take) mee hoon (vermicelli)”….* (P14)

### Behavior to diabetes medication

More than 80% participants (n = 18) were non-adherent to diabetes medications. Lack of disease knowledge was identified from participants’ behavior.

*“(I will) change (my insulin medication) myself. (Although) the doctor has said not to and (if I am) afraid of hypo (glycaemia) I should check (my blood sugar) first, record (my blood sugar levels) and if I continue to be hypo (glycaemic) I should call (the clinic) to reduce (my insulin medication)”…*… (P19)

*“It is not good (anti*-*diabetic medication) because it does not cure but instead worsens (diabetes). The medication keeps increase from half (a dose) to one (dose) to two (doses). Meaning it does not cure but worsens (my condition)”….* (P12)

At the same time, several lay beliefs found to influence the diabetes management. Participants’ lack of awareness towards diabetes treatment showed the possible (Tables 3, 4) cause of non-adherence in the cohort.

**Table 3:** Themes and sub themes of participants

Themes	Probe	Demographics	Responses
Perception towards self-monitoring blood glucose practices			
	In your opinion what is the preferred method of recording blood glucose reading?	59/F Indian Amputate leg	I (prefer to) manually record. I do not understand how to use a telephone especially opening (applications and other function on the telephone). To me manual (recording) is easier
		47/M Malay Amputate arm	I am an old person I like it to be (hand) written. Anyway as long as someone shows me how to do it I can do it (electronic monitoring). Of course it’s easier because you bring your hand phone everywhere you go
		35/F Chinese Amputate leg	(I prefer the) digitals way (telemedicine). Everyday you can see it in your digital way in the software (digital diary) so (there is) no need to record like manually. Sometime(s) even (if) you record manually the paper (is placed) wherever (and will go) missing. (With telemedicine) you have a backup. Due to (limited mobility) I am dependent on family members for (regular check-up), so this electronic log (will help my physician) to track down my performance
	Do you think self-monitoring of blood glucose useful for diabetes management?	44/M Malay Amputate leg	I think, It’s useful to me as an indication (of my sugar control). I prefer that I can use it to check my blood sugar (levels and so I can study how this medication effect(s) my glucose (levels). Also this (reduces my dependency) to family members
		59/F Chinese Amputate arm	Well this is the age of information technology, you can monitor (your health condition) by the Internet everywhere you go. I just (log into) the Internet (and I can track) my dose, what food (or) meal and what nutrition is suitable to my body. You will narrow (down the self-care methods) that suit your body and not other peoples
	What stops people for self-care practices?	46/M Indian Amputate arm	Self-monitoring is okay but sometimes-financial conflict (unable to buy sticks for glucometer) let me forget about checking my sugar for months… then suddenly I few symptoms (hyperglycaemic or hypoglycaemic) pops-up and I remember to continue my sugar monitoring
		55/F Chinese Amputate arm	I think (it) depend(s) on the situation where you live in a village (which is) very difficult now also because certain villages you don’t have traditional gathering so traditional food with rich sugar (often) serve you already (know) that (day) your sugar is not in control… so no point of monitoring. I usually double the drug dose
		63/M Malay Amputate leg	Well what (I can say), I am (afraid) of blood, so I cant monitor (my self) sugar… sometimes my son (when free) check the sugar…… Usually (twice or three) times per month.. but sometimes I feel (dizzy) so I asked him to check (blood sugar)

**Table 4:** Interview key findings on opinion on diet and diabetes management among participants (n = 21)

Themes	Probe	Demographics	Responses
Awareness towards diet management			
	In your opinion what are the strategies to control diet?	34/M Malay Amputate leg	I have my wife (who does the cooking). I’m living in a standard family (of) more than six adult people and more than three children (we) have to cook a lot and then I will have to cook separately
		67/M Chinese Amputate arm	I change everything (diet) because rice is very bad. (I will eat) rice maybe two (to) three time(s) a day (week) only so (instead) I (will take) mee hoon (vermicilli)
	Barriers to controlling diet?	45/M Indian Amputate leg	It is not hard to control (our diet but) sometimes we (do not want to) waste (food) so we will finish (up any left overs). Sometimes your wife might be stressed at work and (when you) come back and say ‘What is this (kind of food)!” then it will become a big issue. (Do you) understand?
		35/F Malay Amputate arm	If we cook separately) it can affect our relationship (with or families). When I do it like that (insisting on eating healthy food) your (there will be) a rift in your famil(ies) relationship so sometimes we do not follow (our diet) that strictly because dinner time is the only time (for a) family gathering so sometimes we will eat out
	Before you diagnosed (diabetes), have you heard of calorie counting?	35/F Malay Amputate arm	I know (about) the calorie count(ing) such as the nutrition (content), cholesterol (and) calories (are all on the food packet) but because we have been used to taking any (food) we like (it is difficult) when I’ve found out that I have this sickness (diabetes) and I have to start controlling this and that but even so I still feel (like I) want to eat the same food. That’s our attitude
		47/M Chinese Amputate arm	We do not know (about calories) we just eat whatever we fancy regardless how how much calorie is in the food
Behavior to diabetes medication			
	What type of experiences with diabetes medication usually reduces the people adherence?	56/M Malay Amputate arm	(I will) change(my insulin medication) myself. (Although) the doctor has said not to and (if I am) afraid of hypo(glycaemia) I should check (my blood sugar) first, record (my blood sugar levels) and if I continue to be hypo(glycaemic) I should call (the clinic) to reduce (my insulin medication)
		59/F Chinese Amputate arm	It is not good (anti-diabetic medication) because it does not cure but instead worsens (diabetes). The medication keeps increase from half (a dose) to one (dose) to two (doses). Meaning it does not cure but worsens (my condition)
	Do you aware of other beliefs (lay beliefs) in people that influence the diabetes management?	46/M Indian Amputate arm	In the beginning I was worried (when I) took (insulin). He (my friend) told me that (insulin) is made out of swine. When I knew of it I did not want (to take insulin that is made from swine). What happens when (a by product of) swine enters (my) body? How am I going to bathe?
		45/F Malay Amputate leg	Correct there is a lack of (diabetes knowledge among the public). People assume that when he has a chronic disease means that he is waiting to die. We have to change our mentality
	Have you heard of alternative medicines for diabetes?	49/M Malay Amputate leg	Pomegranate juice. (when I) ate that I checked that my blood (pressure) reduced a lot
		53/M Indian Amputate leg	This (balsam apple) if you take it daily (your blood) sugar (levels) will go down
		39/F Malay Amputate arm	Usually you soak ladies finger in the water (overnight) and you drink the water tomorrow morning it will also make the (blood) sugar (levels) go down
		43/F Chinese Amputate arm	That “bile of earth” (*Andgrographis paniculata*) if you take that I can assure (you that) hundred percent your BP (blood pressure) will go down you sugar (will) also go down. In fact I have discussed with my doctor and he agrees. He is a very elderly man (but) he agree(s). But you can only take once week not more than three times (or else) you can not urinate and experience erectile dysfunction

“In the beginning I was worried (when I) took (insulin). He (my friend) told me that (insulin) is made out of swine. When I knew of it I did not want (to take insulin that is made from swine). What happens when (a by product of) swine enters (my) body? How am I going to bathe?”… (P1)

Even patients with high economic status, middle age 35–50 and diabetes history of 5–10 years were influenced towards alternative treatments.

*“Pomegranate juice. (When I) ate that I checked that my blood (pressure) reduced a lot”.*. (P17)

*“This (balsam apple) if you take it daily (your blood) sugar (levels) will go down*”… (P9)

*“Usually you soak ladies finger in the water (overnight) and you drink the water tomorrow morning it will also make the (blood) sugar (levels) go down”…* (P5)

*“That “bile of earth” (Andgrographis paniculata) if you take that I can assure (you that) hundred percent your BP (blood pressure) will go down you sugar (will) also go down. In fact I have discussed with my doctor and he agrees. He is a very elderly man (but) he agree(s). But you can only take once week not more than three times (or else) you can not urinate and experience erectile dysfunction”* …. (P16)

### Cues of action

#### Mobile reminder

Although it is advised that self-monitoring is important for diabetics to control their blood glucose levels but participants have reported limited practice to glucometer and family support remains an important factor to ensure compliance:

*“Long*-*term basis we can do ourselves but (it is) better that someone (to) assist or remind us (to control out blood sugar levels) because I take everything easy so my wife will be my reminder she will remind me to do all this la (controlling diabetes). Even for technology (mobile*-*based) or whatever my wife will be the caretaker and remind (me) what to do and what to eat or not to eat”…* (P11)

#### Diabetes education

Many participants acknowledge that diabetes education is important. Participants were interested to gain new knowledge while some showed initiative to attend diabetes education seminars organized by the local clinics. Some participants provided suggestions on how to better encourage other diabetics to attend diabetes education seminars. Participants suggest that as every diabetic should take the initiative to ensure adequate knowledge is obtained in order to better manage their disease:

*“Because this one (diabetic education) is not compulsory. Hospitals should make (it) compulsory for all patient(s) to attend the classes. Patients should be forced to come (and) attend classes also support groups would be better (and) should be free that will help others to understand about diabetes”…*. (P20)

## Discussion

Self-care practices including self-monitoring of blood glucose has an important role in diabetes management. Several studies have documented the relationship between knowledge and self-care practices including; physical activity and adherence to diet. All of them focused on either general population or type 2 diabetes patients but none of them have ever discussed the practices among physically disabled patient [16–19]. This study explores the patients’ practices and barriers to self-care practices.

Self-management is considered as an important part of diabetes care. Also, knowledge, awareness is the greatest weapon in the fight against diabetes mellitus that might help diabetics to understand disease risks, motivate them to seek proper treatment and care, and set up them to keep the disease under control [20–24].

Several variables influence the glucose metabolism among diabetic population, including weight status, gender, age and type of diabetes (insulin dependent versus non-insulin dependent). Majority of studies target the population between age 45–78 years [4, 7, 10–15] when weight concerns are at least level. However about 66% of this study participants were age <50 years. Also awareness of calorie counting as diet control strategy have never discussed before, thus this study have explore the patients’ awareness to understand the concept of calorie counting in diet modification plan. Usually this behavior overestimated with patients’ response only. Studies have suggested that pharmacist-led intervention model significantly improved patients’ knowledge and practices to dietary modification and physical activities [10–15].

Self-monitoring of blood glucose (SMBG) has been recommended by the American Diabetes Association as a test for monitoring the glycemic status [25].

Educational interventions involving patient participation and collaboration seemed to be more effective than didactic interventions in improving glycemic control. The process of self-management includes the tendency to structure situations and activate resources (self-perception), to accept options for action (self-reflection) and to believe in self-efficacy (self-regulation). Structured programs which mostly combine information, strategies for behavioral changes, and self-management strategies are still needed [26].

Aspects of the process of self-management (structuring the situation and activating resources [self-perception], accepting options for action [self-reflection] and believing in self-efficacy [self-regulation]) which lead to a change in the metabolic profile of patients using blood glucose self-monitoring. SMBG coupled with structured brief counseling provided patients with a tool for taking on more self-control and resulted in an improved outlook on life [27].

The study has found several lay beliefs that influence the treatment outcomes. Patients have also claimed the self-prescribing behavior and also lack of diabetes-disease based knowledge. Scientific literature debated on the use of herbal and natural remedies from last several decades, but patient’s behavior is reflective to functional-knowledge about the disease. Therefore, care-plan must include the elements of disease-knowledge, potential determents that influence the treatment course and patients-participation in treatment planning [10, 13, 15].

### Limitations

The study is limited to patients with help-seeking behavior, clearly there are patients not willing to visit healthcare facilities and live in a hostile environment. The limitation of funding restricted the study to conduct a nationwide survey therefore results of this exploratory study are not truly representative of the entire population. This study has not performed any anthropometric (waist circumference, body mass index etc.) correlation with the patients’ responses thus future directions should focus on behavioral relationship with clinical variables.

## Conclusions

This study had identified lack of diabetes related knowledge among physical disabled patients. Self-care blood glucose monitoring is somehow limited but the use of pharmacist or mobile devices might improve the practices. Also study concluded that patients with physical disability required extensive care and effective strategies to control glucose metabolism. Patients with physical disability should be considered as special population and healthcare professionals focus more on improving patients’ knowledge and behavior than treatment plan.

### Practice implication

1. This study is the first to explore the patients’ behavior and practices to disease management among physically disabled type 2 diabetes mellitus patients.
2. Physical disability and cognitive impairments are the major barriers to achieve optimal glycemic control and medication adherence.
3. Somehow the research community ignored to explore the patients’ behavior to self-care practices and medication adherence with physical disability.
